# Promoting Undetectable Equals Untransmittable in Sub-Saharan Africa: Implication for Clinical Practice and ART Adherence

**DOI:** 10.3390/ijerph17176163

**Published:** 2020-08-25

**Authors:** Nicholas Ekow Thomford, Doreen Mhandire, Collet Dandara, George B. Kyei

**Affiliations:** 1 Division of Human Genetics, Department of Pathology & Institute for Infectious Disease and Molecular Medicine, Faculty of Health Sciences, University of Cape Town, Anzio Road, Observatory, Cape Town 7925, South Africa; doreen.mhandire@uct.ac.za (D.M.); collet.dandara@uct.ac.za (C.D.); 2 School of Medical Sciences, College of Health and Allied Sciences, University of Cape Coast, Cape Coast, Ghana; 3 Department of Virology, Noguchi Memorial Institute for Medical Research, University of Ghana, Legon, Ghana; gkyei@noguchi.ug.edu.gh; 4 Department of Medicine, Washington University School of Medicine, St. Louis, MO 63110, USA

**Keywords:** HIV, U = U, anti-retroviral therapy, clinical practice, sub-Saharan Africa, adherence, PLWHIV

## Abstract

In the last decade, reliable scientific evidence has emerged to support the concept that undetectable viral loads prevent human immunodeficiency virus (HIV). Undetectable equals untransmissible (U = U) is a simple message that everyone can understand. The success of this concept depends on strict adherence to antiretroviral therapy (ART) and the attainment of suppressed viral loads (VLs). To achieve U = U in sub-Saharan Africa (SSA), poor adherence to ART, persistent low-level viremia, and the emergence of drug-resistant mutants are challenges that cannot be overlooked. Short of a cure for HIV, U = U can substantially reduce the burden and change the landscape of HIV epidemiology on the continent. From a public health perspective, the U = U concept will reduce stigmatization in persons living with HIV (PLWHIV) in SSA and strengthen public opinion to accept that HIV infection is not a death sentence. This will also promote ART adherence because PLWHIV will aim to achieve U = U within the shortest possible time. This article highlights challenges and barriers to achieving U = U and suggests how to promote the concept to make it beneficial and applicable in SSA. This concept, if expertly packaged by policy-makers, clinicians, health service providers, and HIV control programs, will help to stem the tide of the epidemic in SSA.

## 1. Introduction

Since the discovery of the HIV, the search for effective treatment and cure has occupied the scientific community. Until the discovery of the first antiretroviral (ARV) drug, patients diagnosed with AIDS had between six and 24 months to live. Chronicles of the search to eliminate the virus indicate that the first “ray of hope” in the form of the drug azidothymidine (AZT) was approved by the United States Food and Drugs Administration (FDA) for treating AIDS in 1987 [1]. For over a decade, AZT brought a lifeline to persons living with HIV (PLWHIV) as it reduced the HIV viral loads (VLs) and delayed progression to full-blown AIDS [2,3].

The limitations of a single regimen rapidly became apparent due to the emergence of drug resistance. Initial therapeutic strategies targeted single stages in the viral life cycle. As single regimen faced challenges, there was the need to find innovative ways to improve viral suppression and promote quality of life for patients. The advent of opportunistic infections, viral replication, and drug resistance ushered in combination therapies that were found to be more effective than single therapies such as AZT [4,5]. Intensification in HIV research helped with understanding how the virus integrated itself into host cells and hijacked the host mechanism to replicate itself. Further research findings gave birth to the development of more drugs for ART, targeting different stages of the virus life cycle. The major innovation in HIV management to date is the use of combination ART. Evidence has pointed to the use of combinations of three (sometimes two) ARVs to substantially arrest viral replication [6,7]. These combination therapy strategies resulted in viral suppression to below detectable levels for prolonged and persistent periods in a significant proportion of patients. Triple ART has resulted in a decrease in HIV-associated morbidity and mortality, such that HIV-infected individuals now can live as long a life as their uninfected counterparts.

The sustained ability of ART to keep the VL at undetectable levels has given birth to the concept of undetectable equals untransmissible (U = U). This U = U concept is supported by strong scientific evidence which is showing that consistent undetectable VLs for at least six months may prevent an individual from transmitting HIV sexually [8,9,10]. The U = U concept is also further supported by results showing decreased mother to child transmission (MTCT) from as high as 33% in the pre-ART era to less than 3% in 2010 [11]. With the introduction of Option B+ (lifelong ART for all HIV-infected women regardless of CD4 T cell count and VL) which has more potent viral suppression, the rate of MTCT of HIV has further decreased to <1% even in high-HIV-burden sub-Saharan African (SSA) countries such as Botswana [12]. Evidence exists to show that high maternal VL is the biggest driver of vertical transmission of HIV. Studies carried out in mother–infant cohorts have confirmed low or no MTCT in mothers with low and undetectable VLs.

Given this strong scientific evidence, there have been massive campaigns to promote strict adherence to “test and treat” regardless of CD4 count [13,14]. Following all this evidence, the director of the National Institute of Allergy and Infectious Disease (NIAID), Dr. Anthony S. Fauci and his colleagues stated that “The science does verify and validate undetectable equals untransimissible” [15].

This is best summarized as “The U = U concept bridges the best of the biomedical science with current concepts in behavioral and social science by removing the fear and guilt that a person may be harming someone else, as well as the feeling of self-imposed and external stigma that many people with HIV experience”—RW Eisinger, CW Dieffenbach and AS Fauci (Source: NIAID).

We believe that the evidence is strong enough for SSA countries to promote U = U. This paper reviews the science underlying the U = U concept, identifies potential barriers to promoting it in Africa, and offers suggestions that could help clinicians, policymakers, and other stakeholders to promote the idea on the continent.

## 2. U = U: The Concept, the Science

### 2.1. Low Viral Load and Transmissibility: The Evidence Supporting U = U

The evidence that low VLs result in low transmissibility has been gathered over the last two decades, although the mechanism is yet to be elucidated. An earlier study in the year 2000 which involved the Rakai cohort of Uganda observed that 51 heterosexual HIV seropositive individuals with VLs of <1500 copies/mL did not transmit or infect their partners [9]. In the PARTNER study in 2014–2016, which involved about 14 European countries, authors discovered that for couples who reported <200 copies/mL, there was zero (0) transmission of HIV to seronegative partners who were serodiscordant couples after several exposures to sex without a condom [16]. The latest study in 2018, which is a follow-up of the PARTNER study in gay couples, also confirmed zero transmission when VLs were <200 copies/mL. Other key clinical studies have demonstrated that adequate adherence to ART significantly reduces the risk of HIV transmission to seronegative individuals [10,17]. The HPTN052 was the key study that cemented U = U as concept in SSA. This real-world study undertaken in Malawi, Zimbabwe, South Africa, Botswana, Kenya, Thailand, India, Brazil, and the United States showed ~97% reduced transmission from those whose virus was suppressed [10]. The opposites attract study among Australians, Thais, and Brazilian serodifferent gay couples in 2017 also showed that undetectable VLs (<200 copies/mL) lead to zero transmission [17]. These studies and reports demonstrated that for transmissibility to be possible, at least a VL of 1500 copies/mL coupled with other factors such as co-infections must come into play. Though these studies did elucidate on the ART combinations being administered, that notwithstanding, reduction of VLs prevented transmission in serodiscordant individuals. Studies have also shown low or no MTCT transmission among women with low and undetectable VL compared to those with detectable and high VLs.

### 2.2. Transcriptional Silencing

It is therefore scientifically established that effective combination and adherence to ART efficiently block viral replication in the host leading to a reduction in circulating virus to undetectable levels by standard clinical assays. Though the VLs may remain undetectable, research has shown that there is a small population of resting CD4+ T-cells in the peripheral blood and tissues that harbor replication-competent proviruses [18,19,20]. These proviruses have been found to hide in CD4+ T-cells in anatomical sanctuary sites where they may not readily be accessible to current ART. These sanctuaries include but are not limited to the gut- and rectal-associated lymphoid tissue, lymph node, macrophages, central nervous system, and genital tract [21,22,23]. The most widely accepted reason for viral rebound after ART interruption is the idea of “reactivation from latency”. Here, it is assumed that transcriptionally silent proviruses are reactivated when resting T cells undergo antigen stimulation [24]. This process is ongoing, but under ART, no viral rebound is possible because new T cells are not infected. Over the past few decades, the mechanisms involved in transcriptional silencing have been elucidated in the quest to eradicate HIV. It is now becoming clear that the latent pool of replication-competent proviruses in resting CD4+ T-cells is established very early in the infection process, much larger than previously assumed, can undergo clonal expansion, and decays very slowly with ART. Further, recent research is showing that macrophages, whose role in latency was previously debated, could be an essential source of virus in patients on ART [25,26,27]. These are formidable obstacles to HIV cure [28]. While waiting for a cure, the promotion of U = U is a viable way to turn around the HIV epidemic in Africa.

### 2.3. Clinical Significance of ART Induced Transcriptional Silencing, U = U

The clinical significance of HIV transcriptional silencing amounts to a reduction in new infections of susceptible cells and seronegative individuals as the location, and viral quantities facilitate cell to cell infectivity and serodiscordant transmission. The use of ART is influential enough to halt all new infection of susceptible cells. The decay rate of free circulating viruses is very fast [29] due to the attenuation of the viral life cycle from the ART regimens, which lead to decreased viral plasma levels. Adherence to ART leads to significant reduction in plasma viremia which “forces” some of the viruses to seek refuge in the already mentioned sanctuaries through several yet to be understood mechanisms in transcriptional silencing. Clinical studies have discovered that viremia can fall to as low as <50 copies/mL and remain indefinitely as residual viremia [30,31,32] so long as ART is maintained.

## 3. HIV Clinical Practice in Sub-Saharan Africa and Its Challenges

HIV clinical practice has come a long way in SSA since the discovery of HIV over three decades ago. In SSA, most health facilities have HIV clinics that see PLWHIV, and this also includes mobile clinics that go around to give healthcare to rural or disadvantaged communities [33,34,35]. HIV clinical practice with ART sets to achieve specific ultimate goals, which eventually results in the optimal well-being of the patient (Box 1).

Box 1 **Ultimate goals of HIV treatment.**

To cure, and get rid of the virus

To achieve optimal and persistent VL suppression

Reestablishment and safeguarding immune function

Reducing adverse drug effects and ART-drug/herb-interaction

Preventing transmission or seroconversion

Increasing life expectancy for PLWHIV and improve quality of life

Reducing opportunistic HIV-related morbidity and mortality

With the fall in prices of ART allowing for scaling up of treatment in SSA, the burden of HIV in terms of morbidity and mortality has decreased significantly over the past few decades [36,37]. HIV clinical practice and support systems must be tailored to support the 90-90-90 WHO goals by 2020. Implementing the target of the third 90 requires at least 90% of patients on ARTs to have a VL of <1000 copies/mL to be considered virally suppressed according to the Joint United Nations Programme on HIV/AIDS (UNAIDS) [38]. Between 2000 and 2016, sub-Saharan countries significantly improved their policies to increase the number of PLWHIV with suppressed VLs as low as <400 copies/mL [39]. However, based on high-quality evidence and published studies [40], achieving viral suppression to U = U in SSA needs to be taken further to achieving and maintaining undetectable levels of less than 200 copies/mL. A positive step is a success in implementing policy change and in SSA, which has translated into tremendous decreases in MTCT. There is, however, the need to monitor new policies so that Africa can achieve the Sustainable Development Goal (SDG) of ending the AIDS epidemic by 2030 [41].

The biggest ray of hope in achieving this SDG is by maintaining undetectable VLs for U = U, thus curbing further transmission of HIV. However, access to CD4 monitoring, VL determination, and in some cases ART itself have remained a challenge in some part of SSA. CD4 monitoring is progressively being relegated to the background with less focus on CD4 in ART treatment programs [42]. As a matter of fact, funding agencies for HIV treatment in SSA countries are no longer dedicating funds for CD4 monitoring. The result is that some national programs are abandoning the practice. Although VL monitoring is required to establish virologic suppression, CD4 count is required to make crucial clinical decisions, especially for patients with advanced HIV. These patients require intense follow-up to screen, monitor, and treat opportunistic infections such as cryptococcal diseases and tuberculosis. Therefore, scraping CD4 measurements is likely to lead to increased mortality in PLWHIV in Africa [43]. In our opinion, at least one CD4 test should be done at diagnosis for clinicians to identify those with low CD4 counts.

Over the years, several ARVs have been developed to help to achieve the goals of HIV therapy. Over 50 ARVs have been approved by the US FDA to treat HIV. These drugs target various stages of the HIV life cycle and have proven to be effective when given as combination therapy. The drug options available in SSA are quite limited compared to what is out there in the developed world [44,45]. Despite the introduction of newer drugs which are almost universally accessible in the developed world, most of SSA continues to use the traditional first-line options consisting in tenofovir/lamivudine (TDF/3TC) or tenofovir/emtricitabine (TDF/FTC) and efavirenz (EFV). In some cases, patients are kept on first-line ART despite adverse events and/or clinical failure because of lack of resources or new options.

In SSA, first-line treatment in most HIV clinics is that approved by the FDA. For example, drugs like EFV and TDF are common in the regimen used for treatment. The implementation of the “test and treat” policy where HIV treatment guidelines have been expanded to include persons with HIV regardless of CD4 cell count or clinical stage [46] will aid in achieving U = U. Where first-line treatments fail (TDF/3TC or TDF/FTC and EFV), second-line treatments such as AZT/3TC (or FTC) as the nucleoside reverse transcriptase inhibitor backbone, combined with dolutegravir (DTG) (or atazanavir/ritonavir or lopinavir/ritonaviror darunavir/ritonavir) [46], should be used according to WHO guidelines to aid in achieving U = U. Indeed, where available, this guideline promotes inhibitor DTG combination regimens. The test and treat strategy has been fully embraced in nearly all SSA countries and thus needs to be encouraged as part of efforts to end HIV on the continent [47,48].

The success rates for maintaining viral suppression to undetectable levels in individuals infected with HIV will significantly increase if the newer agents that are potent and well-tolerated are introduced in HIV clinical practice in SSA. Therefore, the effort being made to introduce the integrase inhibitor DTG combination regimens in Africa is a step in the right direction. Sustained efforts must be made to increase patient compliance and avoid the emergence of resistance against these new regimens. Key to achieving undetectable levels includes patients adhering to their ART regimen and maintaining a healthy lifestyle. The success story of U = U in SSA hinges on consistent ART adherence.

## 4. Factors Affecting Adherence to ART in Sub-Saharan Africa

For U = U to be successful in SSA, barriers that affect adherence should be significantly reduced. These barriers present in both structural and clinical forms, leading to poor adherence. Some of the barriers include access to potent and well-tolerated ART, drug stock-outs, stigma, pill burden, and lack of community support for continued adherence [49,50]. The benefits of ART use can be observed if patients focus on long-term commitments to taking their treatment as prescribed. Poor adherence to treatment regimen which can be linked to both patients and healthcare providers has been associated with multidrug resistance, lack of viral suppression, and increase in opportunistic infections [51,52]. SSA. These barriers remain a hindrance to achieving viral suppression to undetectable levels.

However, the continued existence of structural barriers such as economic, institutional and cultural factors influence the way PLWHIV adhere to their medications [50]. Individual-level barriers to ART adherence identified in SSA also include lower levels of education, problems with schedules and routine, stigma and discrimination, side effects, and travel [53,54,55]. Recently, in South Africa, stigma, unemployment, non-disclosure, and alternate forms of therapy have been identified as significant barriers to ART adherence with suggestions that interventional measures to address these barriers will increase adherence [56,57]. Religion has also contributed to a lack of adherence to ART in Africa; in some instances, patients are promised miracle cures. The emergence of prophets who claim to cure HIV has misled some PLWHIV who have then stopped taking ART [58].

Clinical barriers influencing strict adherence to ART include less tolerated ARVs and drug–drug/herb interactions (DDI/DHI) that lead to adverse drug reactions (ADR), discouraging patients from continuing their medication [59]. Pill burden where patients take at least three different types of ARTs serves as one key barrier to adherence [60]. To overcome this barrier, especially in SSA, pill burden can be reduced by providing a simpler dosing regimen (one single tablet per day) to encourage adherence. Polypharmacy complicates adherence, since DDI causes adverse effects and prevents patients from sticking to their regimens. The issue of polypharmacy-related adverse effects is complicated by the use of herbal medications in SSA where these are easily accessible [61,62,63]. The use of other substances such as alcohol and illicit drugs while on ART regimen promotes non-adherence for many patients [64,65]. Traditional complementary and alternative medicine (TCAM) practice, which is highly prevalent in SSA, includes herbal medicine and faith healing methods, including spiritual practices and prayer, and plays a “key” role in HIV treatment. The use of TCAM promotes poor adherence to ART [66,67,68]. Pharmacogenetics, which has resulted in patients reacting differently to the same drug dosage, has also resulted in lack of adherence. This was mostly witnessed with EFV, where a considerable number of patients developed central nervous system related ADRs [69,70]. The ENCORE study and many others have provided overwhelming evidence on how pharmacogenetics suggest personalized prescription of drugs [71,72,73,74,75]. Pharmacogenetics testing has already been implemented for abacavir in the developed world [76,77]. Due to constrained resources, implementing pharmacogenetics in SSA is still to be realized [78].

These barriers which promote non-adherence in SSA countries present significant challenges to achieving U = U in the sub-region. Efforts to break barriers to ART non-adherence will be a much-needed nudge to achieve U = U in PLWHIV in SSA.

## 5. Promoting U = U in Sub-Saharan Africa and the Consequences for Controlling HIV

The epidemiology of HIV in SSA is widely known, with associated challenges in reducing and potentially ending the pandemic. It is now scientifically proven and well accepted in the HIV community that virally suppressed individuals cannot transmit the virus to others. The U = U campaign has been aptly promoted since 2017 and has been endorsed by several organizations and countries, including the United States Centre for Disease Control and Prevention [79]. In SSA countries, the U = U campaign/slogan is gradually being integrated into HIV prevention promotion, although a more vigorous campaign strategy is needed. For PLWHIV, sustained viral suppression with a VL of <200 copies/mL, plus adherence to ART can lead to the maintenance of VLs and zero transmission [80]. Promoting strategies for U = U in SSA countries needs better communication and behavioral commitments [81] (Box 2). Active promotion of U = U in SSA has tremendous benefits for combating HIV in African countries, and overcoming barriers such as those mentioned previously will be a positive step (Box 3). Effective U = U through accessibility, adherence, and monitoring could result in zero (0) new HIV cases.

Box 2 **Communication and behavioral strategies that can be used in promoting U = U in SSA.**

- Clinicians and healthcare providers should discuss U = U with PLWHIV
- Translating of U = U campaign into local dialect as SSA countries have several languages
- Health facilities in SSA countries should have posters (pictorial and words) at HIV clinics to constantly remind patients of U = U
- Community engagement promotion strategies such as display of U = U campaign banners and posters in community facilities that people often visit
- Local and opinion leaders as champions and ambassadors of U = U
- Radio and television adverts
- Social media use

The common barriers to non-adherence in SSA previously highlighted remain a challenge to effectively promoting U = U. It has been revealed that irrespective of the duration of suppression, non-adherence even for a period of 72 h can lead to viral rebound [81]. This makes it pivotal to package the U = U campaign message so that it is succinct, with the recipients understanding that U = U does not equate to a cure. To overcome some of the barriers, we have suggested some interventions which can help to achieve U = U in SSA.

Box 3 **Potential interventions to barriers to undetectable equals untransmissible (U = U).**

**Barriers to U = U**

**Possible Interventions**

Access to potent and well-tolerated ARTDrug stock-outsStigmaLack of community support for continued adherenceCo-infections and comorbidities

ART optimization, reducing cost of ART, SSA countries obtaining new agent class drugsFocused efforts to produce in country WHO standard ARTsDemystifying HIV and AIDS through simple communication strategiesReducing stigma through HIV and AIDS demystificationOptimized therapies to reduce DDI effects

The above interventions can be validated with careful monitoring and record keeping of persons leaving with HIV.

## 6. Conclusions

Governments in SSA countries should increase the number of treatments through the test and treat strategy to bring HIV under control. Continuous access to VL and CD4 monitoring should go hand in hand. This will assure viral suppression and early detection of virologic failure and resistance and help to identify those with low CD4 count who need particular interventions. Bringing U = U promotion to the local level in SSA countries will involve innovative campaign strategies. U = U has consequences in HIV clinical practice in SSA, as it can influence adherence to ART and potentially decrease seroconversion and prevalence. Health ministries in SSA countries should initiate policy changes that will promote U = U in HIV clinical practice. U = U can be made useful in SSA with a combined clinical and social approach.
